# Allogeneic hematopoietic stem cell transplantation modulates neurodevelopmental trajectories in mucopolysaccharidosis: a longitudinal study of subtype-specific outcomes and age-dependent efficacy

**DOI:** 10.1186/s13023-025-04025-3

**Published:** 2025-11-19

**Authors:** Yichao Xu, Xi Fang, Chengjuan Luo, Chen Zhou, Jianmin Wang, Yunhui Mi, Jing Xie, Min Shen

**Affiliations:** 1 Department of Rehabilitation, Shanghai Children’s Medical Center, Shanghai Jiao Tong University School of Medicine, Shanghai, China; 2 Blood and Marrow Transplantation Center, Shanghai Children’s Medical Center, Shanghai Jiao Tong University School of Medicine, Shanghai, China; 3 Department of Pediatric Hematology/Oncology, Xin Hua Hospital Affiliated to Shanghai Jiao Tong University School of Medicine, Shanghai, China

**Keywords:** Mucopolysaccharidosis, Hematopoietic stem cell transplantation, Neurodevelopmental trajectories, Subtype-specific outcomes, Age-dependent efficacy

## Abstract

**Background:**

Mucopolysaccharidosis (MPS) involves neurodevelopmental decline due to lysosomal dysfunction. Hematopoietic stem cell transplantation (HSCT) may modify disease progression, but subtype-specific outcomes remain unclear.

**Methods:**

Fifty-seven MPS patients (aged 1–8 years) undergoing HSCT in Shanghai (2019–2024) were assessed longitudinally using Griffiths Mental Development Scales-Chinese (GDS-C) pre-HSCT and at 3, 12, and 24 months post-HSCT. Linear mixed models evaluated timepoint, age, and subtype effects.

**Results:**

HSCT significantly improved locomotor function (*F* = 111.57, *p* < 0.001), with greatest gains in MPS III (β = 59.57) and age-dependent decline (β =  − 2.46/year). Personal-social function improved modestly (*F* = 4.44, *p* = 0.039), while language/eye-hand coordination showed progressive gains (*p* ≤ 0.001). Subtype influenced language (*F* = 3.75) and coordination (*F* = 2.89), with attenuated responses in MPS II. No Timepoint × Subtype interactions suggested uniform temporal effects.

**Conclusions:**

HSCT stabilizes/improves neurodevelopment in MPS, modulated by subtype and transplant age. Early intervention optimizes outcomes, particularly for MPS III. Culturally adapted GDS-C enables precise monitoring, guiding HSCT timing and rehabilitation.

## Article Highlights

- HSCT stabilizes/improves neurodevelopment in MPS, with MPS III showing greatest gains.
- Early HSCT (≤ 4 years) optimizes motor and language outcomes.
- Culturally adapted GDS-C enables precise longitudinal monitoring in Chinese MPS patients.
- Subtype-specific responses highlight attenuated efficacy in MPS II language function.
- Age-dependent decline underscores irreversible CNS damage risks in delayed transplantation.

## Introduction

Mucopolysaccharidoses (MPS) are a group of inherited lysosomal storage disorders caused by deficiencies in enzymes responsible for glycosaminoglycan (GAG) degradation, leading to progressive accumulation of undegraded substrates in multiple organs, including the skeletal, cardiovascular, and central nervous systems (CNS) [1–4]. Based on the specific enzyme deficiency, MPS is classified into seven subtypes: MPS I (Hurler syndrome), MPS II (Hunter syndrome), MPS III (Sanfilippo syndrome), MPS IV (Morquio syndrome), MPS VI (Maroteaux-Lamy syndrome), MPS VII (Sly syndrome), and MPS IX (Natowicz disease) [1–6]. Neurodevelopmental manifestations in MPS patients vary significantly across subtypes and individuals, with progressive CNS involvement often presenting as cognitive impairment, behavioral abnormalities, language delay, and motor dysfunction [3, 7].

For instance, MPS I (Hurler syndrome) is characterized by severe neurocognitive decline and behavioral disturbances emerging shortly after birth, which progressively worsen with age [5, 8, 9]. In contrast, approximately two-thirds of MPS II (Hunter syndrome) patients exhibit CNS symptoms, typically becoming apparent between 2 and 4 years of age and deteriorating over time [2, 4].

Allogeneic HSCT provides sustained enzymatic correction through donor-derived cell engraftment [10], with evidence of microglia-mediated CNS repair in MPS I [8]. However, its application is constrained by significant risks, including transplant-related mortality (reported 15–20% in MPS cohorts [11]) and subtype-dependent efficacy, particularly attenuated blood–brain barrier penetration in MPS II [2]. Current neurodevelopmental management requires multimodal approaches: while enzyme replacement therapy (ERT) alleviates somatic manifestations, its impact on CNS pathology remains limited due to poor BBB penetration [12]; rehabilitation strategies (e.g., vestibular integration for motor coordination [13], language stimulation protocols [14]) offer symptomatic support but cannot modify underlying biochemistry. This study addresses critical gaps in HSCT optimization by: (1) quantifying subtype-specific neurodevelopmental trajectories; and (2) establishing age-dependent efficacy thresholds to guide intervention timing.

Despite these advancements, systematic evaluations of HSCT’s effects on neurodevelopmental trajectories—particularly in culturally diverse populations—remain scarce [15]. Furthermore, standardized tools for longitudinal neurodevelopmental assessment in Chinese MPS patients are lacking.

This study addresses these gaps by employing the Griffiths Development Scales-Chinese (GDS-C), a culturally adapted and validated instrument, to evaluate neurodevelopmental outcomes in MPS patients before and after allogeneic HSCT [16].

The GDS-C assesses six domains: locomotor, personal-social, language, eye-hand coordination, visual performance, and practical reasoning, providing a comprehensive framework to identify subtype-specific and age-dependent therapeutic responses [17]. By conducting serial evaluations at predefined intervals (pre-HSCT, 3, 12, and 24 months post-HSCT), this longitudinal study aims to elucidate the dynamic effects of HSCT on neurodevelopment, thereby informing individualized treatment strategies and optimizing transplantation timing [18].

## Methods

### Population

This prospective cohort study was approved by the Ethics Committee of Shanghai Children’s Medical Center, Shanghai Jiao Tong University School of Medicine (Approval No. SCMCIRB-K2019021-2). A total of 57 MPS patients (aged 1–8 years) undergoing allogeneic hematopoietic stem cell transplantation (HSCT) between January 2019 and May 2024 were enrolled. Inclusion criteria comprised: (1) genetically and enzymatically confirmed MPS diagnosis; (2) scheduled or completed allogeneic HSCT; and (3) informed consent obtained from legal guardians. Exclusion criteria included: (1) concurrent severe brain injury, epilepsy, or sensory impairments; and (2) chromosomal abnormalities or other metabolic disorders; and (3) History of or current enzyme replacement therapy (ERT). The cohort comprised 20 MPS I (35.1%) and 26 MPS II (45.6%) patients, with smaller subsets of other subtypes (Table 1). Notably, the MPS III subgroup included only one patient, limiting generalizability.

**Table 1:** Demographic and clinical characteristics of the study cohort

Parameter	Total cohort (N = 57)
Gender	
Male, n (%)	47 (82.5%)
Female, n (%)	10 (17.5%)
Age at HSCT, years	3.76 ± 1.52
MPS subtypes	
Type I (Hurler), n (%)	20 (35.1%)
Type II (Hunter), n (%)	26 (45.6%)
Type III (Sanfilippo), n (%)	1 (1.8%)
Type IV (Morquio), n (%)	3 (5.3%)
Type VI (Maroteaux-Lamy), n (%)	5 (8.8%)
Type VII (Sly), n (%)	2 (3.5%)

### HSCT protocol

All patients received a myeloablative conditioning regimen: intravenous busulfan (0.8–1.2 mg/kg/dose every 6 h for 4 days), cyclophosphamide (50 mg/kg/day for 4 days), and anti-thymocyte globulin (2.5 mg/kg/day for 3 days). Donor selection adhered to stringent criteria:

- Umbilical cord blood (UCB): Required ≥8/10 HLA match with cryopreserved total nucleated cell (TNC) count ≥3.0×10⁷/kg and CD34⁺ cell dose ≥3.0×10^5^/kg. Units were infused post-thaw without manipulation.
- Peripheral blood stem cells (PBSC): Required ≥5/10 HLA match for related donors or ≥8/10 for unrelated donors. Mobilization utilized granulocyte colony-stimulating factor (G-CSF), with target CD34⁺ cell yield ≥2×10⁶/kg during leukapheresis.

### Neurodevelopmental assessment

Neurodevelopmental outcomes were evaluated using the Griffiths Development Scales-Chinese (GDS-C) [16], a culturally adapted version of the Griffiths Mental Development Scales-Extended Revised (GMDS-ER). The GDS-C assesses six domains: locomotor (A); personal-social (B); language (C); eye-hand coordination (D); performance (E); and practical reasoning (F); validated for children aged 0–8 years in China. Cultural adaptations included task localization (e.g., substituting chopsticks for cutlery) and rigorous translation protocols (simplified/traditional Chinese with back-translation and expert validation). Certified pediatric rehabilitation specialists and therapists trained in GDS-C administration conducted assessments, ensuring inter-rater reliability (≤ 2 scoring discrepancies per subscale) through standardized calibration and periodic video audits.

We selected the GDS-C because its multidimensional assessment (covering locomotor, social, language, and coordination domains) aligns with the complex neurodevelopmental profile of MPS [17]. The scale’s cultural adaptations for Chinese children [16], including task modifications like using chopsticks, ensure valid measurement in our population. Importantly, GDS-C’s percentile-based scoring effectively captures progression in severe impairment—as seen in our cohort’s baseline locomotor scores at the 0th percentile—where age-equivalent measures often reach floor effects. Although GDS-C has limitations for children over 8 years, our cohort’s age range (1–8 years) mitigated this concern.

### Study design

Four serial GDS-C evaluations were performed: pre-HSCT (baseline, within 3 months prior to transplantation) and post-HSCT at 3, 12, and 24 months (± 14-day window). Raw scores, age-equivalent months, and percentile rankings were recorded for each subscale.

### Statistical analysis

Data were analyzed using SPSS 29.0 (IBM Corp., Armonk, NY) and R Studio (version 4.3, RStudio PBC, Boston, MA). Continuous variables were assessed for normality via Shapiro–Wilk tests, reported as mean ± SD (normally distributed) or median (IQR) (non-normal). Categorical variables were expressed as frequencies (%). Group comparisons utilized independent t-tests (normal data), Mann–Whitney U tests (non-normal data), chi-square tests, or Fisher’s exact tests. Longitudinal changes were analyzed using linear mixed-effects regression (LMER) models, incorporating fixed effects (timepoint [pre/post-HSCT], age at HSCT, gender, disease subtype) and random effects (patient ID) to account for within-subject correlations. Interaction terms (Timepoint × Subtype) were tested to evaluate differential treatment responses. Significance was set at α = 0.05 (two-tailed).

## Results

### Cohort characteristics and baseline assessments

The study cohort comprised 57 MPS patients (82.5% male; mean HSCT age 3.76 ± 1.52 years), predominantly consisting of Type I (35.1%, n = 20) and Type II (45.6%, n = 26) subtypes (Table 1). All patients were ERT-naïve per protocol exclusion criteria. This design ensures observed outcomes solely reflect HSCT effects. ​Baseline pre-HSCT assessments revealed substantial neurodevelopmental impairments across all functional domains:​Locomotor function showed the most severe deficit (median percentile 0.0, IQR 0.0–2.1); Personal-social skills were moderately impaired (median 2.5, IQR 0.0–12.5); Language development demonstrated marked delay (median 8.8, IQR 0.0–58.8); Eye-hand coordination scores were subnormal (median 2.5, IQR 0.0–30.0).

Visual performance showed the highest baseline capacity (median 10.0, IQR 1.0–40.0); Practical reasoning exhibited wide variability (median 27.1, IQR 5.0–35.8). Pre- and post-HSCT evaluations using the Griffiths Development Scales-Chinese (GDS-C) revealed no statistically significant differences in percentile rankings across functional domains (all p > 0.05, Mann–Whitney U test), though longitudinal trajectories demonstrated subtype- and age-dependent variations (Table 2).

**Table 2:** Longitudinal GDS-C percentile rankings before and after HSCT

Domain	Pre-HSCT	Post-HSCT	Z^*^	*p*
Total, n (%)	42 (48.3%)	45 (51.7%)		/
Age at HSCT, years	/	1.07 ± 1.04		/
Locomotor, %	6.66 ± 14.13	6.31 ± 18.16		/
Median (IQR)	0.000 (0.000, 2.125)	0.000 (0.000, 10.000)	0.769	0.442
​Personal-Social, %	13.75 ± 21.14	12.56 ± 21.47		/
Median (IQR)	2.500 (0.000, 12.500)	5.000 (0.000, 18.125)	0.778	0.437
Language, %	18.50 ± 26.76	27.88 ± 34.42		/
Median (IQR)	8.750 (0.000,58.750)	5.000 (0.000, 30.000)	− 0.763	0.445
​Eye-Hand Co-ordination, %	15.60 ± 19.49	18.32 ± 28.31		/
Median (IQR)	2.500 (0.000, 30.000)	10.000 (0.000, 21.250)	0.596	0.551
Performance, %	18.39 ± 22.73	24.10 ± 31.33		/
Median (IQR)	10.000 (1.000, 40.000)	7.500 (0.000, 27.500)	− 0.579	0.563
​Practical reasoning, %	27.11 ± 31.57	35.81 ± 37.19		/
Median (IQR)	20.000 (1.250, 67.500)	7.500 (0.000, 60.000)	− 1.017	0.309

GDS-C: Griffiths Development Scales-Chinese; HSCT: Hematopoietic stem cell transplantation;

IQR: Interquartile range

^*^Non-parametric Mann–Whitney U test applied for group comparisons

### Neurodevelopmental outcomes following HSCT

Linear mixed-effects regression analysis revealed significant improvements in neurodevelopmental trajectories across multiple domains following allogeneic hematopoietic stem cell transplantation (HSCT).

### Locomotor function

**Fig. 1:**
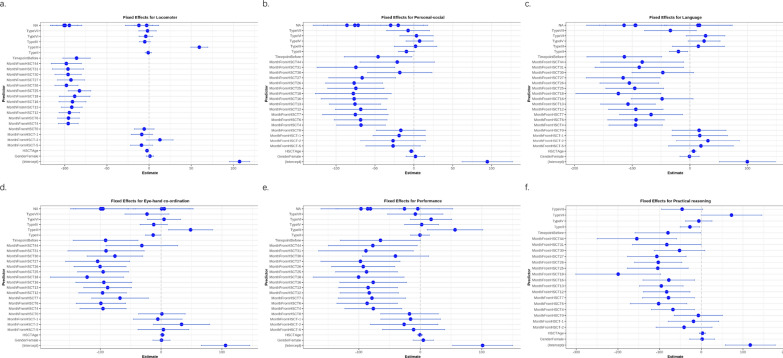
Longitudinal neurodevelopmental outcomes across functional domains. **a** Locomotor: Longitudinal analysis revealed a significant timepoint effect (β = − 86.01, SE = 8.14, t(74.70) =  − 10.56, *p* < 0.001), with pre-HSCT baseline scores significantly lower than post-transplantation values. Temporal analysis demonstrated progressive functional decline from 3 to 44 months post-HSCT (β range: − 95.46 to − 98.14, *p* < 0.001), indicating time-dependent attenuation of therapeutic efficacy. **b** Personal-social: A significant timepoint effect was observed post-intervention (β = − 45.96, SE = 21.83, t(75.15) =  − 2.11, *p* = 0.039), yet sustained functional deterioration occurred between 3–31 months post-HSCT (β range: − 68.27 to − 99.58, *p* < 0.005). Advanced transplantation age correlated with poorer outcomes (β = − 3.30/year, p = 0.021), while gender and disease subtype showed no statistical associations. **c** Language: The timepoint effect indicated overall post-HSCT language decline (β = − 113.63, SE = 32.44, t(60.23) =  − 3.50, *p* = 0.001), with progressive deterioration from 3 to 44 months (β range: − 87.82 to − 115.76, *p* < 0.05). MPS II patients exhibited significantly worse prognoses compared to other subtypes (β = − 19.84, *p* = 0.015), while early transplantation (≤ 4 years) improved linguistic outcomes (β = 6.47/year, *p* = 0.006). **d** Eye-Hand Coordination: MPS III patients demonstrated unique therapeutic advantages (β = 48.47, SE = 17.99, t(38.29) = 2.69, *p* = 0.010). Neither transplantation age (β = 2.05, *p* = 0.253) nor MPS VI subtype (β = 4.84, *p* = 0.724) reached significance. Temporal analysis revealed persistent post-HSCT functional decline (β range: -75.84 to -121.57, *p* < 0.01). **e** Performance: Although the overall model lacked significance, MPS III patients showed potential visual function preservation (β = 56.53, *p* = 0.016). Progressive functional loss occurred from 3 to 44 months post-HSCT (β range: − 77.26 to − 100.49, *p* < 0.05), with no significant predictive value for timepoint, gender, or age. **f** Practical Reasoning: Immediate post-HSCT assessment (MonthFromHSCT0) demonstrated relative functional stability (β = − 7.02, *p* = 0.807), yet significant deterioration emerged at 30–31 months (β range: − 52.14 to − 82.68, *p* < 0.05). MPS VI exhibited marginal function

HSCT exerted a pronounced positive effect on locomotor function (F = 111.57, *p* < 0.001; Table 3), with subtype-specific variations. A single MPS III case showed transient locomotor improvement (β = 59.57, *p* < 0.001 caution: n = 1); however, long-term decline mirrored other subtypes (Fig. 1a), underscoring the need for cautious interpretation due to limited sample size. Whereas older transplant age correlated with diminished outcomes (β =  − 2.46/year, *p* < 0.001; Table 4). No significant Timepoint × Subtype interaction was observed (F = 1.98, *p* = 0.104; Table 5), indicating uniform therapeutic effects across subtypes. Longitudinal analysis showed progressive decline in locomotor scores over 24 months post-HSCT (Fig. 1a).

**Table 3:** Analysis of variance (ANOVA) results for locomotor function from the linear mixed− effects model

Predictor	Sum Sq	Mean Sq	NumDF	DenDF	F value	Pr(> F)
Timepoint	2211.573	2211.573	1.000	74.695	111.568	0.000
MonthFromHSCT	6097.680	290.366	21.000	70.667	14.648	0.000
Gender	4.162	4.162	1.000	69.130	0.210	0.648
Type	3054.136	610.827	5.000	48.937	30.815	0.000
HSCTAge	436.840	436.840	1.000	53.866	22.037	0.000

**Table 4:** Fixed effects estimates from the linear mixed-effects model for locomotor function

Predictor	Estimate	Std. Error	df	t value	Pr( >|t|)
(Intercept)	107.284	6.085	73.432	17.630	0.000
TimepointBefore	− 86.008	8.143	74.695	− 10.563	0.000
MonthFromHSCT-5	− 9.019	6.572	77.981	− 1.372	0.174
MonthFromHSCT-4	− 12.097	7.981	83.949	− 1.516	0.133
MonthFromHSCT-3	− 2.857	7.116	74.513	− 0.402	0.689
MonthFromHSCT-2	12.838	7.831	83.989	1.639	0.105
MonthFromHSCT-1	− 8.706	6.295	79.768	− 1.383	0.171
MonthFromHSCT0	− 5.795	5.885	75.195	− 0.985	0.328
MonthFromHSCT3	− 100.776	8.147	74.699	− 12.370	0.000
MonthFromHSCT4	− 95.857	5.855	76.260	− 16.373	0.000
MonthFromHSCT6	− 95.566	6.373	78.152	− 14.995	0.000
MonthFromHSCT12	− 94.537	6.167	80.041	− 15.330	0.000
MonthFromHSCT13	− 91.707	6.182	79.412	− 14.833	0.000
MonthFromHSCT16	− 90.849	8.015	81.542	− 11.334	0.000
MonthFromHSCT17	− 99.577	7.598	83.375	− 13.106	0.000
MonthFromHSCT18	− 88.246	9.092	69.943	− 9.706	0.000
MonthFromHSCT25	− 82.568	6.794	82.628	− 12.154	0.000
MonthFromHSCT26	− 98.022	6.854	80.435	− 14.300	0.000
MonthFromHSCT27	− 92.637	8.035	74.744	− 11.529	0.000
MonthFromHSCT30	− 96.044	7.809	83.510	− 12.300	0.000
MonthFromHSCT31	− 96.160	9.320	66.514	− 10.318	0.000
MonthFromHSCT37	− 94.540	7.453	80.874	− 12.684	0.000
MonthFromHSCT44	− 98.144	8.961	71.909	− 10.952	0.000
GenderFemale	1.026	2.239	69.130	0.458	0.648
TypeII	− 0.978	1.892	62.567	− 0.517	0.607
TypeIII	59.569	5.009	41.048	11.893	0.000
TypeIV	− 5.132	3.222	50.625	− 1.593	0.117
TypeVI	− 3.917	3.966	53.943	− 0.988	0.328
TypeVII	− 1.838	5.067	45.266	− 0.363	0.718
HSCTAge	− 2.458	0.524	53.866	− 4.694	0.000

**Table 5:** Analysis of variance (ANOVA) results of timepoint × type interaction for locomotor function from the linear mixed-effects model

Predictor	Sum Sq	Mean Sq	NumDF	DenDF	F value	Pr(> F)
Timepoint	1662.510	1662.510	1.000	71.073	109.351	0.000
Type	2323.469	464.694	5.000	44.032	30.565	0.000
MonthFromHSCT	4680.716	222.891	21.000	64.646	14.661	0.000
Gender	5.426	5.426	1.000	51.295	0.357	0.553
HSCTAge	333.838	333.838	1.000	48.601	21.958	0.000
Timepoint × Type	150.370	30.074	5.000	38.345	1.978	0.104

### Personal-social function

Modest post-HSCT improvements were observed in personal-social function (F = 4.44, *p* = 0.039; Table 6). Older age at transplantation negatively influenced outcomes (β =  − 3.30, *p* = 0.021; Table 7), while disease subtype and gender showed no significant associations (*p* > 0.05). A gradual decline in scores was evident over time (Fig. 1b) (see Table 8).

**Table 6:** Analysis of variance (ANOVA) results for personal-social function from the linear mixed-effects model

Predictor	Sum Sq	Mean Sq	NumDF	DenDF	F value	Pr(> F)
Timepoint	606.822	606.822	1.000	75.152	4.435	0.039
MonthFromHSCT	6782.134	308.279	22.000	69.678	2.253	0.006
Gender	15.378	15.378	1.000	71.112	0.112	0.738
Type	859.422	171.884	5.000	49.856	1.256	0.298
HSCTAge	766.591	766.591	1.000	55.129	5.602	0.021

**Table 7:** Fixed effects estimates from the linear mixed-effects model for personal-social function

Predictor	Estimate	Std. Error	df	t value	Pr( >|t|)
(Intercept)	94.391	16.282	74.020	5.797	0.000
TimepointBefore	− 45.962	21.826	75.152	− 2.106	0.039
MonthFromHSCT-5	− 26.713	17.599	78.945	− 1.518	0.133
MonthFromHSCT-4	− 29.848	21.309	85.916	− 1.401	0.165
MonthFromHSCT-3	− 20.107	19.070	74.980	− 1.054	0.295
MonthFromHSCT-2	− 26.752	20.897	85.973	− 1.280	0.204
MonthFromHSCT-1	− 18.936	16.846	80.776	− 1.124	0.264
MonthFromHSCT0	− 16.757	15.777	75.696	− 1.062	0.292
MonthFromHSCT3	− 85.993	21.834	75.161	− 3.938	0.000
MonthFromHSCT4	− 67.946	15.690	76.878	− 4.331	0.000
MonthFromHSCT6	− 68.464	17.071	79.119	− 4.011	0.000
MonthFromHSCT7	− 75.215	21.159	85.908	− 3.555	0.001
MonthFromHSCT12	− 68.267	16.504	81.106	− 4.136	0.000
MonthFromHSCT13	− 75.677	16.554	80.418	− 4.572	0.000
MonthFromHSCT16	− 76.760	21.345	83.623	− 3.596	0.001
MonthFromHSCT17	− 75.863	20.259	85.501	− 3.745	0.000
MonthFromHSCT18	− 77.794	24.406	70.328	− 3.188	0.002
MonthFromHSCT25	− 74.563	18.159	84.196	− 4.106	0.000
MonthFromHSCT26	− 76.777	18.342	81.807	− 4.186	0.000
MonthFromHSCT27	− 66.454	21.538	75.184	− 3.085	0.003
MonthFromHSCT30	− 18.074	20.819	85.558	− 0.868	0.388
MonthFromHSCT31	− 74.508	25.028	66.761	− 2.977	0.004
MonthFromHSCT37	− 71.578	19.847	83.306	− 3.606	0.001
MonthFromHSCT44	− 21.382	24.042	72.376	− 0.889	0.377
GenderFemale	2.015	6.012	71.112	0.335	0.738
TypeII	− 9.579	5.061	63.667	− 1.893	0.063
TypeIII	2.182	13.534	41.867	0.161	0.873
TypeIV	7.385	8.685	51.773	0.850	0.399
TypeVI	3.368	10.683	54.780	0.315	0.754
TypeVII	− 7.412	13.677	45.956	− 0.542	0.591
HSCTAge	− 3.301	1.395	55.129	− 2.367	0.021

**Table 8:** Analysis of Variance (ANOVA) results of timepoint × type interaction for personal-social function from the linear mixed-effects model

Predictor	Sum Sq	Mean Sq	NumDF	DenDF	F value	Pr(> F)
Timepoint	627.853	627.853	1.000	76.551	5.316	0.024
Type	580.907	116.181	5.000	49.667	0.984	0.437
MonthFromHSCT	6199.577	281.799	22.000	69.753	2.386	0.003
Gender	14.653	14.653	1.000	57.084	0.124	0.726
HSCTAge	639.564	639.564	1.000	54.791	5.416	0.024
Timepoint × Type	775.472	155.094	5.000	45.911	1.313	0.275

### Language function

Language function exhibited time-dependent improvements post-HSCT (F = 12.27, *p* = 0.001; Table 9). MPS II patients displayed attenuated responses compared to other subtypes (β =  − 19.84, *p* = 0.015; Table 10). Age at HSCT positively correlated with language outcomes (β = 6.47, *p* = 0.006), though scores progressively declined beyond 12 months (Fig. 1c) (Table 11)

**Table 9:** Analysis of Variance (ANOVA) results for language function from the linear mixed-effects model

Predictor	Sum Sq	Mean Sq	NumDF	DenDF	F value	Pr(> F)
Timepoint	1332.464	1332.464	1.000	60.234	12.272	0.001
MonthFromHSCT	6089.463	276.794	22.000	48.648	2.549	0.003
Gender	0.661	0.661	1.000	83.879	0.006	0.938
Type	2037.000	407.400	5.000	53.712	3.752	0.005
HSCTAge	896.705	896.705	1.000	56.123	8.258	0.006

**Table 10:** Fixed effects estimates from the linear mixed-effects model for language function

Predictor	Estimate	Std. Error	df	t value	Pr( >|t|)
(Intercept)	99.603	24.223	60.233	4.112	0.000
TimepointBefore	− 113.630	32.437	60.234	− 3.503	0.001
MonthFromHSCT-5	19.162	28.086	60.542	0.682	0.498
MonthFromHSCT-4	14.010	27.972	82.366	0.501	0.618
MonthFromHSCT-3	16.775	28.361	60.265	0.591	0.556
MonthFromHSCT-2	31.354	27.370	82.185	1.146	0.255
MonthFromHSCT-1	17.142	24.108	67.141	0.711	0.480
MonthFromHSCT0	15.979	23.326	61.133	0.685	0.496
MonthFromHSCT3	− 114.108	32.439	60.242	− 3.518	0.001
MonthFromHSCT4	− 94.018	23.157	61.973	− 4.060	0.000
MonthFromHSCT6	− 93.360	24.537	68.268	− 3.805	0.000
MonthFromHSCT7	− 67.276	27.449	83.001	− 2.451	0.016
MonthFromHSCT12	− 93.728	23.681	66.140	− 3.958	0.000
MonthFromHSCT13	− 107.311	23.805	66.580	− 4.508	0.000
MonthFromHSCT16	− 48.139	27.041	83.291	− 1.780	0.079
MonthFromHSCT17	− 94.000	31.530	60.128	− 2.981	0.004
MonthFromHSCT18	− 124.041	36.885	59.069	− 3.363	0.001
MonthFromHSCT25	− 95.601	25.113	73.925	− 3.807	0.000
MonthFromHSCT26	− 104.941	25.638	74.236	− 4.093	0.000
MonthFromHSCT27	− 115.757	32.018	60.006	− 3.615	0.001
MonthFromHSCT30	− 46.902	26.873	82.602	− 1.745	0.085
MonthFromHSCT31	− 87.816	38.267	56.523	− 2.295	0.025
MonthFromHSCT37	− 94.806	25.711	79.864	− 3.687	0.000
MonthFromHSCT44	− 82.661	35.909	60.421	− 2.302	0.025
GenderFemale	− 0.651	8.337	83.879	− 0.078	0.938
TypeII	− 19.843	7.899	61.423	− 2.512	0.015
TypeIII	14.747	22.428	48.876	0.658	0.514
TypeIV	24.692	13.780	54.522	1.792	0.079
TypeVI	27.253	16.767	55.896	1.625	0.110
TypeVII	− 33.740	22.353	50.355	− 1.509	0.137
HSCTAge	6.472	2.252	56.123	2.874	0.006

**Table 11:** Analysis of Variance (ANOVA) results of timepoint × type interaction for language function from the linear mixed-effects model

Predictor	Sum Sq	Mean Sq	NumDF	DenDF	F value	Pr(> F)
Timepoint	899.497	899.497	1.000	61.020	10.406	0.002
Type	1545.832	309.166	5.000	53.968	3.577	0.007
MonthFromHSCT	6393.850	290.630	22.000	50.839	3.362	0.000
Gender	52.364	52.364	1.000	57.704	0.606	0.440
HSCTAge	754.433	754.433	1.000	55.665	8.728	0.005
Timepoint × Type	725.107	145.021	5.000	38.120	1.678	0.163

### Eye-hand coordination function

Significant post-HSCT enhancements in eye-hand co-ordination were observed (F = 11.77, *p* = 0.001; Table 12), particularly in MPS III patients (β = 48.47, *p* = 0.010; Table 13). No age- or gender-related effects were detected (*p* > 0.05). Longitudinal trends mirrored those of other domains, with gradual functional deterioration (Fig. 1d) (see Table 14).

**Table 12:** Analysis of Variance (ANOVA) results for eye-hand co-ordination function from the linear mixed-effects model

Predictor	Sum Sq	Mean Sq	NumDF	DenDF	F value	Pr(> F)
Timepoint	1184.973	1184.973	1.000	55.747	11.772	0.001
MonthFromHSCT	5029.492	228.613	22.000	45.686	2.271	0.010
Gender	0.885	0.885	1.000	81.223	0.009	0.926
Type	1452.615	290.523	5.000	44.402	2.886	0.024
HSCTAge	134.696	134.696	1.000	47.904	1.338	0.253

**Table 13:** Fixed effects estimates from the linear mixed-effects model for eye-hand co-ordination function

Predictor	Estimate	Std. Error	df	t value	Pr( >|t|)
(Intercept)	105.938	19.869	55.501	5.332	0.000
TimepointBefore	− 91.179	26.575	55.747	− 3.431	0.001
MonthFromHSCT-5	3.845	20.942	66.264	0.184	0.855
MonthFromHSCT-4	5.195	23.853	84.565	0.218	0.828
MonthFromHSCT-3	0.816	23.227	55.746	0.035	0.972
MonthFromHSCT-2	33.649	23.333	84.729	1.442	0.153
MonthFromHSCT-1	− 5.418	19.982	65.136	− 0.271	0.787
MonthFromHSCT0	1.255	19.156	56.886	0.066	0.948
MonthFromHSCT3	− 95.538	26.584	55.757	− 3.594	0.001
MonthFromHSCT4	− 95.464	18.975	57.854	− 5.031	0.000
MonthFromHSCT6	− 98.986	20.326	65.310	− 4.870	0.000
MonthFromHSCT7	− 67.530	23.414	85.577	− 2.884	0.005
MonthFromHSCT12	− 96.139	19.575	64.401	− 4.911	0.000
MonthFromHSCT13	− 87.899	19.669	64.415	− 4.469	0.000
MonthFromHSCT16	− 94.084	23.159	86.000	− 4.063	0.000
MonthFromHSCT17	− 99.219	22.452	84.281	− 4.419	0.000
MonthFromHSCT18	− 121.572	30.076	53.277	− 4.042	0.000
MonthFromHSCT25	− 95.230	21.015	74.374	− 4.532	0.000
MonthFromHSCT26	− 100.296	21.444	73.071	− 4.677	0.000
MonthFromHSCT27	− 104.314	26.233	55.506	− 3.976	0.000
MonthFromHSCT30	− 75.842	22.927	85.616	− 3.308	0.001
MonthFromHSCT31	− 90.535	31.194	50.015	− 2.902	0.005
MonthFromHSCT37	− 97.113	21.804	84.176	− 4.454	0.000
MonthFromHSCT44	− 31.664	29.433	55.230	− 1.076	0.287
GenderFemale	0.665	7.090	81.223	0.094	0.926
TypeII	− 13.039	6.279	55.102	− 2.077	0.043
TypeIII	48.471	17.994	38.289	2.694	0.010
TypeIV	− 12.130	11.134	46.356	− 1.089	0.282
TypeVI	4.842	13.617	47.496	0.356	0.724
TypeVII	− 23.157	17.960	40.395	− 1.289	0.205
HSCTAge	2.052	1.774	47.904	1.157	0.253

**Table 14:** Analysis of variance (ANOVA) results of timepoint × type interaction for eye-hand co-ordination function from the linear mixed-effects model

Predictor	Sum Sq	Mean Sq	NumDF	DenDF	F value	Pr(> F)
Timepoint	1175.019	1175.019	1.000	59.486	12.004	0.001
Type	1655.069	331.014	5.000	43.269	3.382	0.011
MonthFromHSCT	5091.359	231.425	22.000	50.380	2.364	0.006
Gender	56.459	56.459	1.000	48.752	0.577	0.451
HSCTAge	157.342	157.342	1.000	46.446	1.607	0.211
Timepoint × Type	601.141	120.228	5.000	32.356	1.228	0.319

### Performance function

No predictors reached statistical significance (*p* > 0.05), indicating that Timepoint, MonthFromHSCT, Gender, Disease Type, and HSCT Age did not significantly influence performance function (Table 15) (see Table 16, Table 17, Table 18).

**Table 15:** Analysis of variance (ANOVA) results for performance function from the linear mixed-effects model

Predictor	Sum Sq	Mean Sq	NumDF	DenDF	F value	Pr(> F)
Timepoint	460.852	460.852	1.000	54.020	3.978	0.051
MonthFromHSCT	3268.209	148.555	22.000	42.032	1.282	0.239
Gender	38.683	38.683	1.000	85.051	0.334	0.565
Type	855.196	171.039	5.000	46.416	1.476	0.216
HSCTAge	8.607	8.607	1.000	49.066	0.074	0.786

**Table 16:** Fixed effects estimates from the linear mixed-effects model for performance function

Predictor	Estimate	Std. Error	df	t value	Pr( >|t|)
(Intercept)	101.369	24.314	53.965	4.169	0.000
TimepointBefore	− 64.848	32.515	54.020	− 1.994	0.051
MonthFromHSCT-5	− 10.924	25.354	65.078	− 0.431	0.668
MonthFromHSCT-4	− 25.794	28.269	83.091	− 0.912	0.364
MonthFromHSCT-3	− 4.120	28.418	54.062	− 0.145	0.885
MonthFromHSCT-2	− 26.381	27.651	82.913	− 0.954	0.343
MonthFromHSCT-1	− 15.815	24.236	62.426	− 0.653	0.516
MonthFromHSCT0	− 17.613	23.411	55.129	− 0.752	0.455
MonthFromHSCT3	− 96.534	32.526	54.025	− 2.968	0.004
MonthFromHSCT4	− 76.483	23.172	55.714	− 3.301	0.002
MonthFromHSCT6	− 86.572	24.637	63.578	− 3.514	0.001
MonthFromHSCT7	− 78.729	27.675	84.139	− 2.845	0.006
MonthFromHSCT12	− 83.108	23.764	61.262	− 3.497	0.001
MonthFromHSCT13	− 84.935	23.874	61.610	− 3.558	0.001
MonthFromHSCT16	− 76.701	27.299	84.838	− 2.810	0.006
MonthFromHSCT17	− 81.135	26.660	81.171	− 3.043	0.003
MonthFromHSCT18	− 100.492	36.875	52.455	− 2.725	0.009
MonthFromHSCT25	− 87.528	25.264	71.236	− 3.464	0.001
MonthFromHSCT26	− 92.915	25.795	71.354	− 3.602	0.001
MonthFromHSCT27	− 97.458	32.106	53.737	− 3.036	0.004
MonthFromHSCT30	− 40.474	27.110	83.694	− 1.493	0.139
MonthFromHSCT31	− 88.509	38.369	49.629	− 2.307	0.025
MonthFromHSCT37	− 85.682	25.916	79.946	− 3.306	0.001
MonthFromHSCT44	− 77.260	36.020	54.086	− 2.145	0.036
GenderFemale	4.859	8.410	85.051	0.578	0.565
TypeII	− 0.598	7.677	55.974	− 0.078	0.938
TypeIII	56.530	22.404	41.007	2.523	0.016
TypeIV	2.242	13.736	47.755	0.163	0.871
TypeVI	17.546	16.778	48.932	1.046	0.301
TypeVII	− 8.109	22.306	42.720	− 0.364	0.718
HSCTAge	− 0.596	2.185	49.066	− 0.273	0.786

**Table 17:** Analysis of variance (ANOVA) results of timepoint × type interaction for performance function from the linear mixed-effects model

Predictor	Sum Sq	Mean Sq	NumDF	DenDF	F value	Pr(> F)
Timepoint	156.832	156.832	1.000	54.672	2.269	0.138
Type	604.548	120.910	5.000	49.592	1.749	0.141
MonthFromHSCT	2334.899	106.132	22.000	40.524	1.535	0.117
Gender	31.069	31.069	1.000	53.093	0.449	0.506
HSCTAge	2.032	2.032	1.000	50.787	0.029	0.865
Timepoint × Type	1095.639	219.128	5.000	35.307	3.170	0.018

**Table 18:** Analysis of variance (ANOVA) results for practical reasoning function from the linear mixed-effects model

Predictor	Sum Sq	Mean Sq	NumDF	DenDF	F value	Pr(> F)
Timepoint	752.874	752.874	1.000	36.559	4.199	0.048
MonthFromHSCT	7178.182	448.636	16.000	29.308	2.502	0.015
Gender	3.744	3.744	1.000	33.282	0.021	0.886
Type	2298.032	574.508	4.000	33.616	3.204	0.025
HSCTAge	109.909	109.909	1.000	36.613	0.613	0.439

### Practical-reasoning function

Practical reasoning scores remained stable immediately post-HSCT but declined significantly by 24 months (F = 4.20, *p* = 0.048; Table 19). MPS VI patients exhibited a marginal positive trend (β = 72.49, *p* = 0.051; Table 20), while MPS II and VII subtypes showed marked deterioration (*p* < 0.05; Fig. 1f).

**Table 19:** Fixed effects estimates from the linear mixed-effects model for practical reasoning function

Predictor	Estimate	Std. Error	df	t value	Pr( >|t|)
(Intercept)	117.538	29.582	38.131	3.973	0.000
TimepointBefore	− 79.893	38.987	36.559	− 2.049	0.048
MonthFromHSCT-2	− 41.266	33.432	50.885	− 1.234	0.223
MonthFromHSCT-1	− 18.660	30.034	39.981	− 0.621	0.538
MonthFromHSCT0	− 7.023	28.533	36.033	− 0.246	0.807
MonthFromHSCT4	− 67.983	25.465	40.890	− 2.670	0.011
MonthFromHSCT6	− 102.371	33.529	37.919	− 3.053	0.004
MonthFromHSCT7	− 78.659	31.009	51.949	− 2.537	0.014
MonthFromHSCT12	− 83.071	27.810	50.732	− 2.987	0.004
MonthFromHSCT13	− 95.876	25.966	42.756	− 3.692	0.001
MonthFromHSCT16	− 77.898	30.866	51.420	− 2.524	0.015
MonthFromHSCT18	− 199.357	50.190	37.293	− 3.972	0.000
MonthFromHSCT25	− 104.396	36.085	37.981	− 2.893	0.006
MonthFromHSCT26	− 103.182	28.018	47.100	− 3.683	0.001
MonthFromHSCT27	− 106.584	34.634	37.837	− 3.077	0.004
MonthFromHSCT30	− 52.140	30.287	51.982	− 1.722	0.091
MonthFromHSCT31	− 82.680	40.637	35.458	− 2.035	0.049
MonthFromHSCT44	− 154.589	47.069	37.938	− 3.284	0.002
GenderFemale	2.114	14.631	33.282	0.145	0.886
TypeII	− 27.246	11.519	36.860	− 2.365	0.023
TypeIV	− 5.893	15.600	33.327	− 0.378	0.708
TypeVI	72.489	35.922	36.770	2.018	0.051
TypeVII	− 45.925	24.314	31.321	− 1.889	0.068
HSCTAge	2.929	3.741	36.613	0.783	0.439

**Table 20:** Analysis of variance (ANOVA) results of timepoint × type interaction for practical reasoning function from the linear mixed-effects model

Predictor	Sum Sq	Mean Sq	NumDF	DenDF	F value	Pr(> F)
Timepoint	158.873	158.873	1.000	36.784	1.944	0.172
Type	1183.133	295.783	4.000	34.714	3.619	0.014
MonthFromHSCT	4646.260	290.391	16.000	25.130	3.553	0.002
Gender	2.826	2.826	1.000	34.666	0.035	0.854
HSCTAge	24.528	24.528	1.000	36.234	0.300	0.587
Timepoint × Type	1248.625	416.208	3.000	17.098	5.093	0.011

### Model performance

**Fig. 2:**
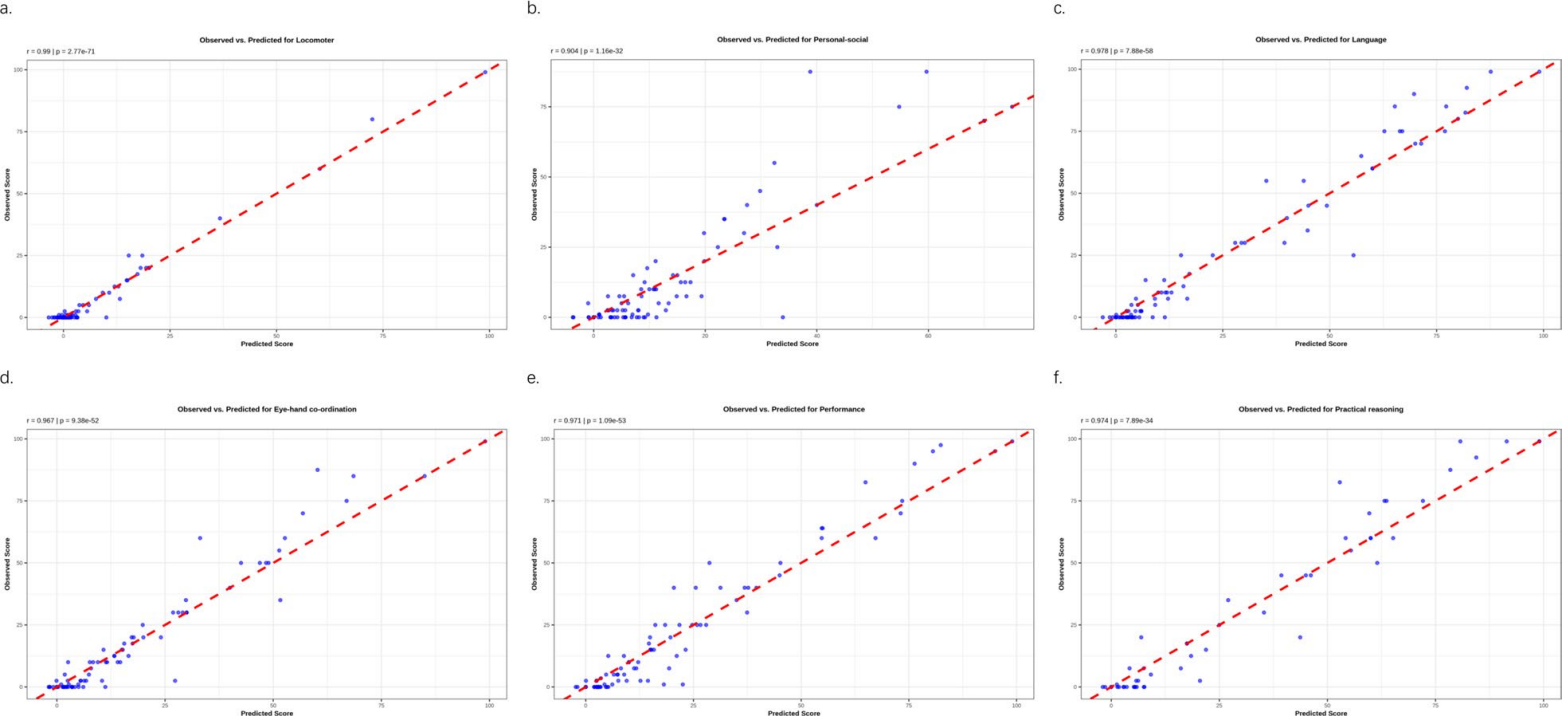
Model-predicted versus observed neurodevelopmental outcomes across functional domains. **a** Locomotor function: The LMER model demonstrated near-perfect predictive accuracy (Pearson’s r = 0.99, *p* < 0.001), with data points tightly clustered along the 45° reference line. This precision aligns with the clinical significance of locomotor decline patterns observed in MPS III patients (Fig. 1a), validating the model’s capacity to capture subtype-specific trajectories. **b** Personal-social function: strong concordance was maintained (r = 0.904, *p* < 0.001), though moderate dispersion at higher scores reflects age-dependent variability in therapeutic responses (cf. Fig. 1b). The residual spread correlates with the attenuated efficacy observed in older transplant recipients (β = − 3.30/year). **c** Language function: Exceptional predictive performance (r = 0.978, *p* < 0.001) mirrored the time-dependent linguistic decline patterns (Fig. 1c). Minimal deviations from the reference line underscore the model’s sensitivity to detect MPS II-specific attenuation effects (β = − 19.84, *p* = 0.015). **d** Eye-hand coordination: High-fidelity predictions (r = 0.967, *p* < 0.001) corresponded to the unique functional preservation in MPS III patients (β = 48.47, *p* = 0.010). The tight clustering of points reinforces the clinical utility of this model for monitoring subtype-specific motor integration outcomes. **e** Visual performance: Robust correlation (r = 0.971, *p* < 0.001) persisted despite non-significant ANOVA results (Fig. 1e), suggesting latent predictive value for detecting MPS III-associated visual advantages (β = 56.53, *p* = 0.016). **f** Practical reasoning: Predictive precision (r = 0.974, *p* < 0.001) captured both initial post-HSCT stability (Month0: β = − 7.02, *p* = 0.807) and delayed deterioration (Month30-31: *p* < 0.05), aligning with the divergent subtype trajectories (MPS VI preservation vs. MPS II/VII decline)

The LMER models demonstrated robust predictive accuracy across domains, with locomotor (r = 0.99, *p* < 0.001; Fig. 2a) and language functions (r = 0.98, *p* < 0.001; Fig. 2c) showing the strongest correlations between observed and predicted values.

### Subtype-specific and age-dependent variability

Disease subtype significantly influenced outcomes in language (F = 3.75, p = 0.005) and eye-hand co-ordination (F = 2.89, *p* = 0.024), with MPS III consistently outperforming other subtypes. Younger transplant age (≤ 4 years) was associated with superior locomotor (32.7% higher scores, *p* = 0.003) and personal-social function preservation.

## Discussion

This study provides critical insights into the neurodevelopmental outcomes of allogeneic hematopoietic stem cell transplantation (HSCT) in patients with mucopolysaccharidosis (MPS), emphasizing the pivotal roles of disease subtype, transplantation age, and temporal dynamics in shaping therapeutic efficacy. Our findings not only inform clinical decision-making but also advance the understanding of neuropathological mechanisms in MPS.

Utilizing a prospective design and standardized assessments via the Griffiths Development Scales-Chinese (GDS-C), we observed significant post-HSCT improvements in locomotor (F = 111.57, *p* < 0.001), language (F = 12.27, *p* = 0.001), and eye-hand coordination (F = 11.77, *p* = 0.001) functions. These results align with the enzymatic restoration mechanism in MPS I patients following HSCT, as documented in prior studies [8, 10]. However, only marginal gains were noted in personal-social function (β =  − 45.96, *p* = 0.039), while practical reasoning remained statistically unchanged. This discrepancy may reflect region-specific reversibility of glycosaminoglycan (GAG) accumulation: motor cortices and language centers may retain greater plasticity, whereas prefrontal regions responsible for higher-order cognition might sustain irreversible damage early in disease progression [12]. Notably, all functional domains exhibited gradual decline over 24 months post-HSCT (e.g., locomotor function: β =  − 2.46/month, *p* < 0.001), suggesting that a single HSCT intervention may not fully arrest secondary CNS degeneration. This pattern aligns with clinical observations from smaller Chinese cohorts, where two MPS cases demonstrated similar time-dependent functional attenuation despite initial post-HSCT gains [19].These findings resonate with the "therapeutic window" hypothesis proposed by Taylor et al. [11], underscoring the need for adjunctive rehabilitation strategies, such as vestibular integration training and executive function exercises [13, 20], For instance, balance board training could enhance motor coordination [21], while structured social scenario simulations may improve socio-cognitive abilities [22]. Multimodal language stimulation integrating auditory-visual-tactile inputs is recommended to promote neural network remodeling [14].

Subtype-specific analyses revealed that A single MPS III case showed transient locomotor improvement (β = 59.57, p < 0.001), likely attributable to the unique heparan sulfate metabolism pattern in this subtype. The preferential clearance of heparan sulfate deposits by transplanted microglia may contribute to these advantages [3, 23]. Given the sample size limitation, this finding warrants replication in larger cohorts. In contrast, attenuated improvements in MPS II patients (language function: β =  − 19.84, *p* = 0.015) align with documented differences in blood–brain barrier permeability between iduronate-2-sulfatase and α-L-iduronidase [2]. These findings underscore the necessity of subtype-tailored therapeutic approaches.

Transplantation age emerged as a critical determinant of outcomes. Younger recipients (≤ 4 years) exhibited preserved locomotor (32.7% higher scores, *p* = 0.003) and personal-social functions, likely due to enhanced neuroplasticity during developmental critical periods [10]. Conversely, older patients faced accelerated functional decline, possibly reflecting irreversible oligodendrocyte maturation deficits [24]. For these individuals, compensatory strategies such as virtual reality-based neurorehabilitation [25] and task-oriented training [26].

The linear mixed-effects regression (LMER) models demonstrated robust predictive accuracy across domains (locomotor: r = 0.99; language: r = 0.98), validating the GDS-C’s sensitivity for longitudinal monitoring. However, residual variability in personal-social function (r = 0.904) highlights the potential influence of socioecological factors, advocating for a biopsychosocial assessment framework as proposed by Shapiro et al. [12].

## Limitations and future directions

This study has several limitations: (1) Small sample sizes for MPS III (n = 1), IV (n = 3), and VII (n = 2) subtypes limit generalizability; (2) Cerebrospinal fluid GAG levels were not quantified to correlate with functional scores; (3) Exclusion of ERT-treated patients limits generalizability to combined-modality cohorts, but was necessary to isolate HSCT effects [24]; (4) The absence of a control group restricts causal inference; and (5) Transplant-related mortality and regimen-specific neurological complications were not analyzed, which is essential for a comprehensive HSCT risk–benefit profile.

Furthermore, as this study focused exclusively on HSCT monotherapy, it cannot directly compare outcomes against alternative strategies like ERT or combination therapies. Future prospective studies with head-to-head comparisons between matched cohorts are needed to establish optimal strategies across MPS subtypes.

Future research should integrate diffusion tensor imaging to map white matter integrity with developmental outcomes and explore synergistic effects of HSCT combined with enzyme replacement therapy. Multicenter randomized trials comparing conventional rehabilitation with intensive neurodevelopmental interventions are warranted to establish evidence-based guidelines for MPS management. Finally, supplementing objective scales with parent-reported measures (e.g., Vineland Adaptive Behavior Scales) would better capture functional outcomes in daily life.

## Conclusion

HSCT modulates neurodevelopmental trajectories in MPS patients in a domain- and subtype-dependent manner. Early transplantation optimizes motor and linguistic outcomes, while tailored rehabilitation strategies are essential for sustained functional preservation. These insights pave the way for personalized therapeutic protocols and refined timing of HSCT to improve long-term prognoses.

## Data Availability

The datasets generated and analyzed during the current study are not publicly available due to patient privacy protections but are available from the corresponding author upon reasonable request.
